# The Role of La_2_O_3_ in Enhancement the Radiation Shielding Efficiency of the Tellurite Glasses: Monte-Carlo Simulation and Theoretical Study

**DOI:** 10.3390/ma14143913

**Published:** 2021-07-13

**Authors:** Aljawhara H. Almuqrin, Mohamed Hanfi, K. G. Mahmoud, M. I. Sayyed, Hanan Al-Ghamdi, Dalal Abdullah Aloraini

**Affiliations:** 1 Department of Physics, College of Science, Princess Nourah bint Abdulrahman University, Riyadh P.O. Box 11671, Saudi Arabia; ahalmoqren@pnu.edu.sa (A.H.A.); hmalghmdi@pnu.edu.sa (H.A.-G.); daalorainy@pnu.edu.sa (D.A.A.); 2 Institute of Physics and Technology, Ural Federal University, St. Mira 19, 620002 Yekaterinburg, Russia; mokhamed.khanfi@urfu.ru; 3 Department of Nuclear Power Plants and Renewable Energy Sources, Ural Power Engineering Institute, Ural Federal University, St. Mira 19, 620002 Yekaterinburg, Russia; kmakhmud@urfu.ru; 4 Department of Nuclear Medicine Research, Institute for Research and Medical Consultations (IRMC), Imam Abdulrahman bin Faisal University (IAU), Dammam P.O. Box 31441, Saudi Arabia

**Keywords:** interactions with photons, glass, simulation, radiation

## Abstract

The radiation shielding competence was examined for a binary glass system xLa_2_O_3_ + (1 − x) TeO_2_ where x = 5, 7, 10, 15, and 20 mol% using MCNP-5 code. The linear attenuation coefficients (*LAC*s) of the glasses were evaluated, and it was found that LT20 glass has the greatest *LAC*, while LT5 had the least *LAC*. The transmission factor (TF) of the glasses was evaluated against thicknesses at various selected energies and was observed to greatly decrease with increasing thickness; for example, at 1.332 MeV, the TF of the LT5 glass decreased from 0.76 to 0.25 as the thickness increased from 1 to 5 cm. The equivalent atomic number (Z_eq_) of the glasses gradually increased with increasing photon energy above 0.1 MeV, with the maximum values observed at around 1 MeV. The buildup factors were determined to evaluate the accumulation of photon flux, and it was found that the maximum values for both can be seen at around 0.8 MeV. This research concluded that LT20 has the greatest potential in radiation shielding applications out of the investigated glasses due to the glass having the most desirable parameters.

## 1. Introduction

Radiation shielding materials used in several industrial and medical fields to absorb radiation currently demand better properties than traditionally used ceramics, concretes, polymers, and composite materials [1,2]. Shielding materials are categorized as any materials used to attenuate incoming radiation to a safe level. Lead-free materials are particularly sought after in dentistry and medicine to minimize the harmful effects of lead and lead composites while using cheap and lightweight shields.

It is well known that ionizing radiation is dangerous since it has enough energy to ionize the medium [3,4]. The incoming radiation’s type and energy affect its strength and properties, but they all give rise to carcinogenesis, cataracts, tissue damage, and cancer. X-rays and gamma rays are two types of radiation that are essential in medical applications for therapeutic and diagnostic purposes [5]. Consequently, it has become necessary to use radiation shields in order to adequately protect humans and mitigate the negative effects of radiation [6,7,8,9]. Thus, reliable and effective materials are important to protect patients’ and workers’ health from exposure to high doses of radiation.

Several previous works have examined, experimentally and theoretically, the use of concretes, glasses, alloys, and ceramics as shielding materials. They evaluated shielding parameters using suitable software programs and simulation codes to assess the effectiveness of the materials for shielding applications [10,11,12,13,14,15]. Lead and other materials that possess a high density are widely used in the medical field; however, lead is toxic to the environment and humans, causing significant side effects. Accordingly, researchers try to solve this problem by using non-toxic materials as radiation shields [16,17,18].

Currently, glasses are commonly used as protective materials against ionizing radiation because of their interesting characteristics, including a relatively low cost, eco-friendliness, ease of preparation, transparency, and easily controllable density during the preparation of the samples, making it possible to obtain glass samples that are effective radiation attenuators [19,20].

Tellurite-based glass systems have received much attention due to their good optical and radiation attenuation characteristics, such as a high dielectric constant, refractive index, thermal stability, density, and effective atomic number [21]. Moreover, the Tellurite-based glasses can be prepared at comparatively low melting temperatures. They have fascinating physical properties such as low phonon energy, excellent chemical endurance, excellent mechanical strength, and high rare-earth ion solubility [22]. On the other hand, La_2_O_3_ is one of the cheapest rare earth oxides, has a band gap of 5.8 eV, and has a large refractive index. Some works have shown that incorporating La_2_O_3_ to tellurite-based glass causes an improvement in the radiation attenuation capabilities. 

Several parameters must be determined to accurately assess a glass’s capability to be used as a suitable shield. The glasses must then be compared with other common materials, such as lead, lead composites, commercial glasses, and many types of heavy concretes. These parameters can be obtained through one of three techniques: theoretical calculations with equations, numerical estimations using simulation codes, and experimental procedures in the lab [23,24].

Among these three techniques, Monte Carlo simulations are one of the essential methods used to evaluate radiation shielding parameters, especially in the absence of the necessary facilities and equipment to carry out practical experiments, as well as the inability to carry out the investigation in research laboratories or universities at present due to the spread of the coronavirus [25]. Accordingly, we applied this simulation technique in this work to report the radiation attenuation features of binary tellurite glass system. The novelty of the present work is studying the influence of the insertion La_2_O_3_ with various concentrations (5, 7, 10, 15, and 20 mol%) on the shielding features of a binary tellurite glass system, as was examined and fabricated by Kaur et al. [26]. Kaur et al. demonstrated that substituting Te_2_O_3_ by La_2_O_3_ increases the fabricated glasses’ density from 5.67 g cm^−3^ to 5.76 g cm^−3^. The investigated glasses’ molecular weight also increased from 167.91 to 192.84 g mol^−1^ when increasing the La_2_O_3_ ratio from 0 to 20 mol%. The Monte Carlo Code (MCNP-5) code was utilized to estimate the investigated glass samples’ attenuation features. The half-value layer (HVL) and mean free path (MFP) were calculated based on the simulated linear attenuation coefficient (*LAC*) results.

## 2. Materials and Methods

**Figure 1:**
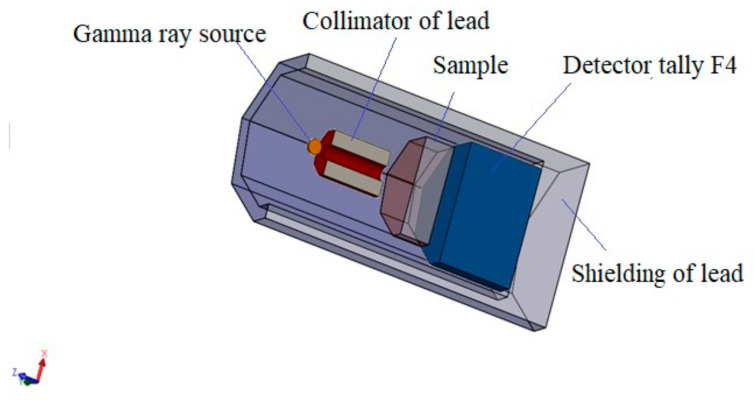
The 3D geometry representing the arranged input file.

In the current investigation, the MCNP-5 code was applied to study the shielding capabilities of lanthanum tellurite (LT) glasses. An input file used for this purpose is illustrated in Figure 1. A 5 cm lead cylinder shielded the detector, the samples, and the source of gamma photons from any external interference. Various gamma sources were selected: ^60^Co with two energies, namely 1.173 and 1.332 MeV, ^137^Cs with one energy value (0.662 MeV), ^228^Th with an energy of 0.24 MeV, and ^214^Bi with an energy of 0.665 MeV. A lead collimator was assumed in the input file to collimate the photons. This collimator has the following dimensions: the height is 10 cm, the central diameter is 2 cm, and the total diameter is 7 cm. The collimated photons then passed via the LT sample, which was positioned between the collimator and the detector [27,28,29]. Table 1 shows the chemical compositions of the LT glasses, and we used these compositions to define the sample into the input file.

The linear attenuation coefficient (*LAC*, cm^−1^) is presented in Equation (1) [22]:

(1) $$LAC=\frac{1}{x}\ln\left(\frac{I_{o}}{I}\right)$$

where *x* refers to the material thickness, $I_{o}$ and I denote the incoming and transmitted intensities.

The mass attenuation coefficient (*MAC*, cm^2^/g) is correlated with the *LAC* by:

(2) $$MAC=\frac{LAC\;}{\rho \;}\;$$

where *ρ* (g/cm^3^) is the fabricated material density. The *ρ* (g/cm^3^) plays a great role in the shielding performance, where the denser the materials, the better and higher the *LAC* achieved. Moreover, the half-value layer (HVL), mean free path (MFP), and transmission factor (*TF*) are decreased with increasing material density.

The transmission factor (*TF*) is also another parameter that can be used to anticipate the proportion of the photons that can transmit any shield with a known thickness. *TF* is simply evaluated using the next Equation (3) [30].

(3) $$TF=\frac{I}{I_{o}}=exp\left(-LAC.x\right)$$

Half-value thickness (HVL, cm) represents the thickness of the shield that can decrease the incoming photon intensity to 50% of its original value. It is mathematically given by Equation (4) [31].

(4) $$HVL=\frac{\ln\left(2\right)}{LAC\;}\;$$

Mean free path (*MFP*, cm) is also considered for the radiation protection study. The number of interactions between the radiation and the shield increases as the distance between the 2 subsequent interactions diminishes, increasing absorption and attenuation. MFP can also be calculated using the *LAC* values, similar to *HVL*, as shown in Equation (5).

(5) $$MFP=\frac{1}{LAC\;}\;\;$$

The BXCOM program was generated to determine the values of other shielding factors like the effective/equivalent atomic numbers (Z_eff_/Z_eq_), as well as the exposure/ energy absorption buildup factors (EBF/EABF) [32].

## 3. Results and Discussion

### 3.1. Linear and Mass Attenuation Coefficients

**Figure 2:**
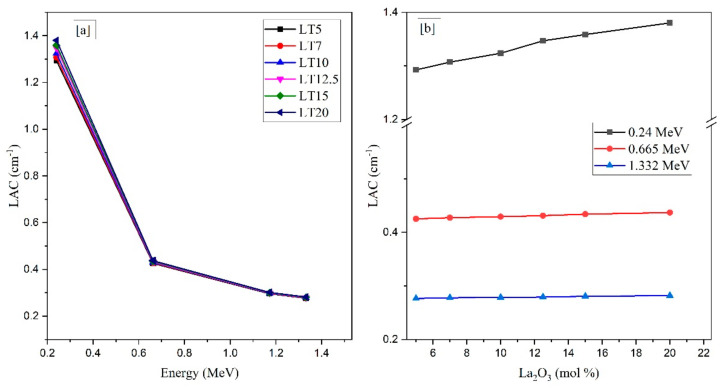
The linear attenuation coefficient (*LAC*) against (**a**) Gamma-ray photon energy and (**b**) La_2_O_3_ content.

MCNP-5 code was used to simulate the *LAC* of the selected LT samples. Figure 2 illustrates that two variables affect the simulated *LAC*. At low energy levels (0.24 MeV specifically), the highest *LAC* was obtained. For the LT5 and LT20 glasses at this energy, the highest *LAC* is equal to 1.29 to 1.38 cm^−1^_,_ respectively. As the energy increases, the simulated *LAC* values progressively decreased. This trend is linked to the Compton scattering (CS) interaction. The lowest *LAC* was obtained at high gamma energies, which increased from 0.276 to 0.282 cm^−1^ for the LT5 and LT20 glasses, respectively.

The *LAC* was affected by incorporating La_2_O_3_ to the selected specimens, where the molecular weight of LT specimens increased and the Z_eff_ decreased. The *LAC* has lower values at low La_2_O_3_ quantities (5 mol % or LT5) while having greater values for the LT20 sample with 20 mol% La_2_O_3_. The *LAC* values decreased in the following order: LT20 > LT15 > LT10 > LT7 > LT5. The *LAC* values for LT5 reduced from 1.29 to 0.276 cm^−1^ and decreased from 1.38 to 0.281 cm^−1^ for the LT20 sample between 0.240–1.332 MeV.

Lanthanum possesses the maximum atomic number among the LT glasses’ chemical components, resulting in the mass density increasing as the molar concentration of La_2_O_3_ increases. A higher mass density is synonymous with tightly packed particles for materials with similar chemical compositions. Thus, it leads to an increase in the radiation interaction cross-sections and thus a higher *MAC*. This present study agrees with the experimental work performed in References [33,34], where they concluded that the addition of HMO has a significant effect in improving the characteristics of various glasses fabricated for radiation shielding purposes.

The *MAC* of the LT glasses was determined using the previous *LAC* data. Additionally, the *MAC* for the LT samples was obtained using XCOM software [35]. The percent difference between the *MAC* values gathered from MCNP5 and XCOM was computed using the following equation:

(6) $$Diff\;\left(\%\right)=\frac{\left[{\left(MAC\right)}_{mcnp}-{\left(MAC\right)}_{xcom}\right]}{{\left(MAC\right)}_{mcnp}}\times 100\;$$

The investigated glasses’ diff % is listed in Table 2 and Table 3, which show that the diff (%) is less than 3% for all the studied LT samples.

### 3.2. The Transmission Factor

**Figure 3:**
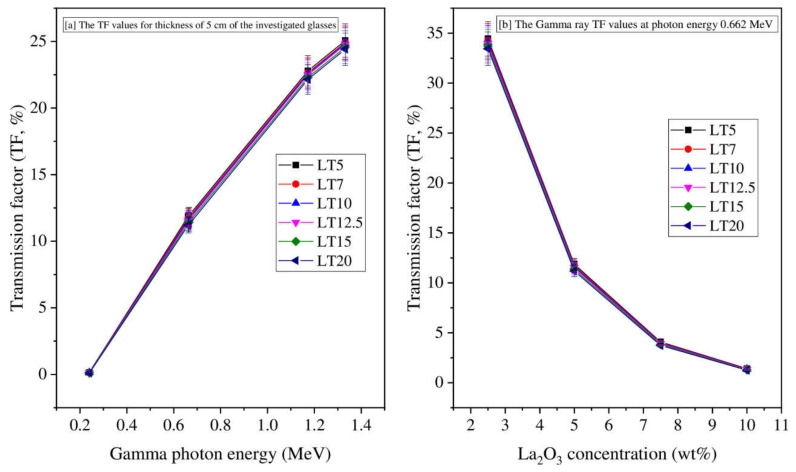
The TF of the LT glasses versus (**a**) Gamma-ray energy and (**b**) the glass thickness.

The behavior of the transmission factor (TF) for the studied glass against glass thickness at five selected energies, namely 0.240, 0.662, 0.665, 1.173, and 1.332 MeV, is illustrated in Figure 3. Two important factors impacted TF. At the lowest examined energy, 0.240 MeV, TF declined from 0.15 to 0.10%. In addition, the highest TF values were obtained at 1.332 MeV, which decreased from 25.07 to 24.42% when the La_2_O_3_ varied between 9.7 to 33.8 wt.%. Therefore, as energy increases, the number of photon interactions within the LT samples decreased, increasing TF. The TF for LT5 glass was reduced from 34% (for x = 2.5 cm) to 1.40% (for x = 10 cm), where x represents glasses’ thickness at 0.662 MeV. The time needed for the photons to pass via the LT specimen increases when the glass thickness increases. Thus, there will be more photon interactions within the material and a reduction in the TF. Consequently, increasing the glass thickness leads to an enhancement in radiation protection.

### 3.3. Half-Value Layer

**Figure 4:**
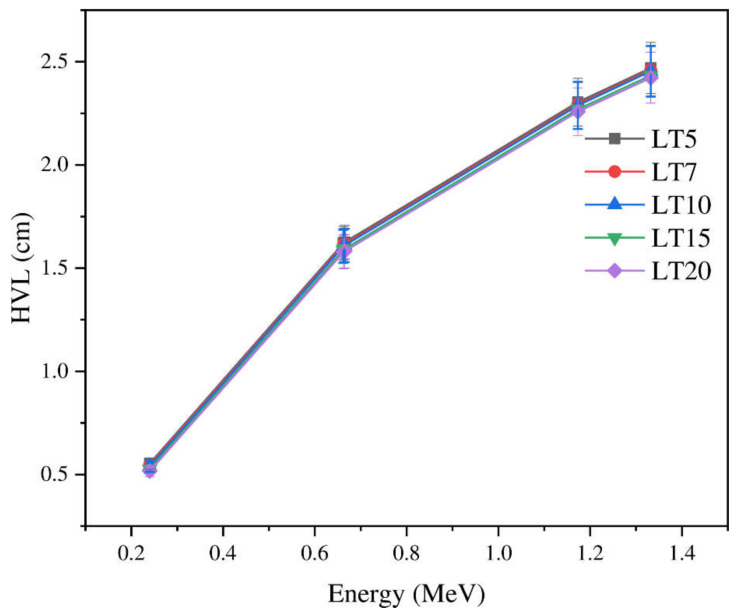
The HVL of the current samples vs. energy for the investigated glass samples with different La_2_O_3_ contents.

**Figure 5:**
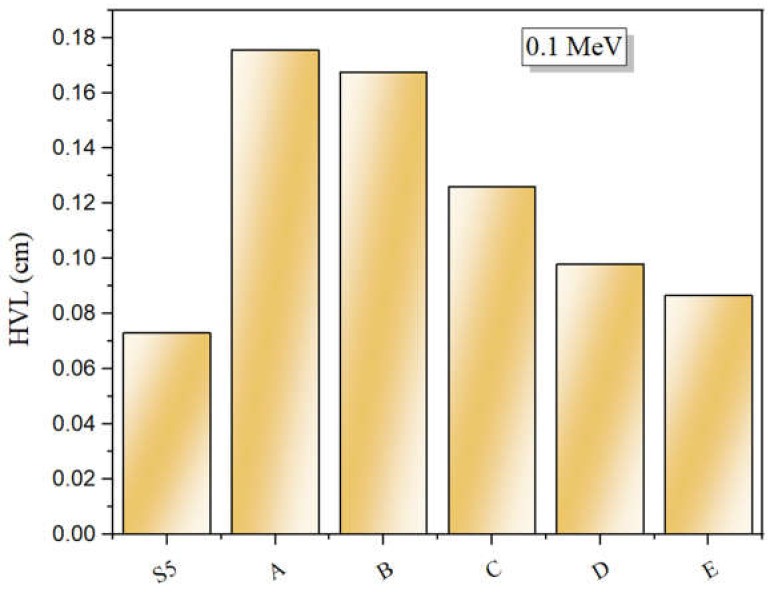
Comparison between the HVL of the investigated glasses and other similar La_2_O_3_ based TeO_2_ glasses.

Figure 4 depicts that HVL, which can be determined from Equation (4). Clearly, increasing the energy of the photons causes a slow increase in the HVL. The HVL values increase from 0.553 to 2.47 cm for LT5 and from 0.516 to 2.42 cm for LT20 between 0.240 and 1.332 MeV. Within the tested energy range (0.240–1.332 MeV), the CS interaction dominates, which means that HVL α E. Subsequently, HVL continues to increase with energy. As illustrated in Figure 4, TeO_2_ content has a notable role in the simulated HVL. Specifically, the addition of TeO_2_ content progressively reduces the HVL. For LT5 and LT20 glasses, the HVL decreased from 0.553 to 0.516 cm. The glasses’ molecular weight and density also increased with the addition of TeO_2_ to the La_2_O_3_ glass samples. Moreover, the HVL of the LT glasses decreased when *LAC* and Z_eff_ increased. In Figure 5, we compare the HVL for LT20 at 0.1 MeV with other TeO_2_ glasses doped with La_2_O_3_ reported in Reference [22]. Evidently, LT20 possesses a lower HVL than the glasses A–E.

### 3.4. Mean Free Path

**Figure 6:**
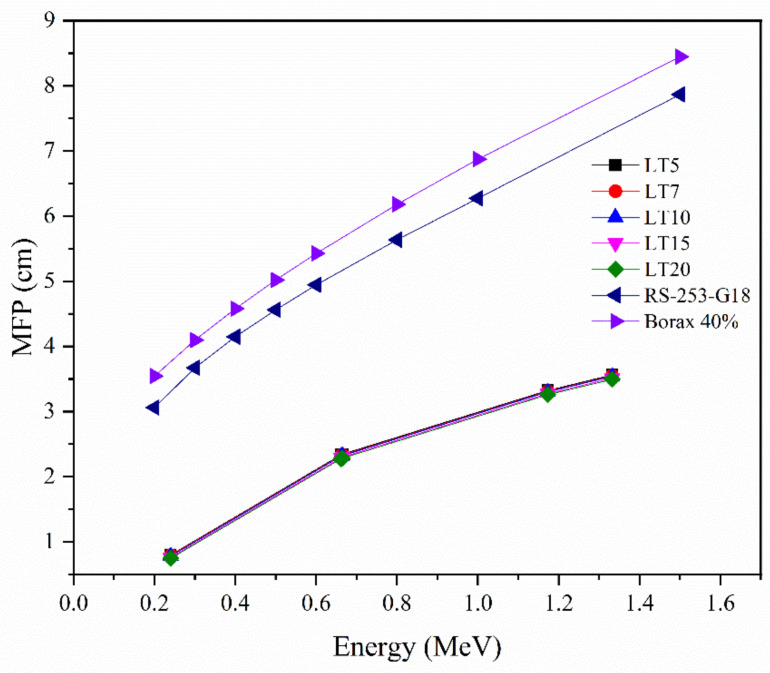
The MFP of the current LT sample compared with RS-253-G18 and Borax 40% glass.

Figure 6 shows the MFP of the LT5-LT20 samples in the chosen energies, and the values were compared with Borax glass [36] and RS-253-G18 [37] commercial shielding glass. Out of the LT samples, the LT20 glass had the maximum MFP value, whereas the minimum value was reported for the LT5 sample. Nevertheless, the MFP of all the LT glasses is lower than the MFP values for Borax 40% and RS-253-G18, proving their capability as a high-density protection glass. These results demonstrate that the average distance between subsequent collisions in the studied LT glasses is relatively small.

### 3.5. Effective and Equivalent Atomic Number

**Figure 7:**
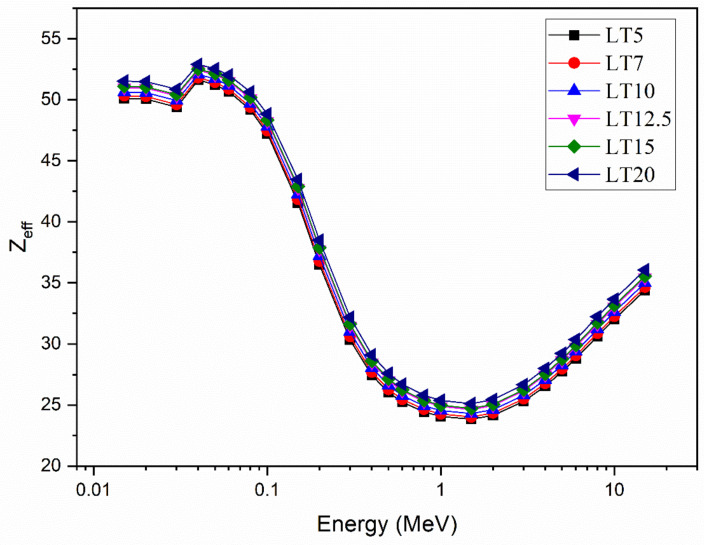
The effective atomic number (Z_eff_) of the LT samples at the tested photon energy zone.

The Z_eff_ results for the current glass systems are illustrated in Figure 7. Three different interactions can be observed within the selected gamma energy ranges. First, the photoelectric effect (PE) appears at low energies (0.015–1 MeV). As energy increases, the Z_eff_ values can be seen to drop. Because of the K-absorption edge of Te, a spike in the Z_eff_ values occurs at 0.0318 MeV. Above 0.1 MeV, the estimated Z_eff_ moderately decreases with energy because of the dominance of the CS interaction and the CS cross-section σ_CS_ α E^−1^. Z_eff_ starts to increase with the growth of energy above several MeV. 

**Figure 8:**
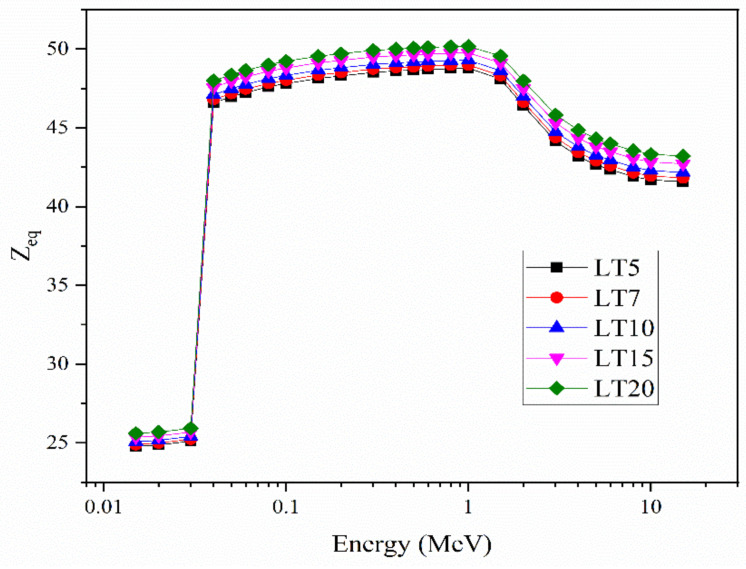
The equivalent atomic number for the LT samples.

The Z_eq_ was computed for *MAC*_CS_. The change in Z_eq_ with the energy is demonstrated in Figure 8. Evidently, the lower Z_eq_ values are found in the PE region. The Z_eq_ values gradually increased with energy greater than 0.1 MeV, where the CS interaction is dominant (>0.1–1 MeV). The maximum Z_eq_ values were observed at around 1 MeV. When the PP interactions increased, Z_eq_ moderately decreased.

### 3.6. Buildup Factors

One of the most important shielding parameters describes the material’s ability to absorb or accumulate the photon flux during exposure to a radioactive source, namely the glasses’ buildup factors: the exposure/energy absorption buildup factors (EBF/EABF). 

The geometric progression was determined, and the EBF and EABF were theoretically estimated for the LT5-LT20 specimens using BXCOM. The program’s calculations were computed between 0.015 and 15 MeV for some penetration depths starting at 0.5 mfp and continuing to 20 mfp. 

**Figure 9:**
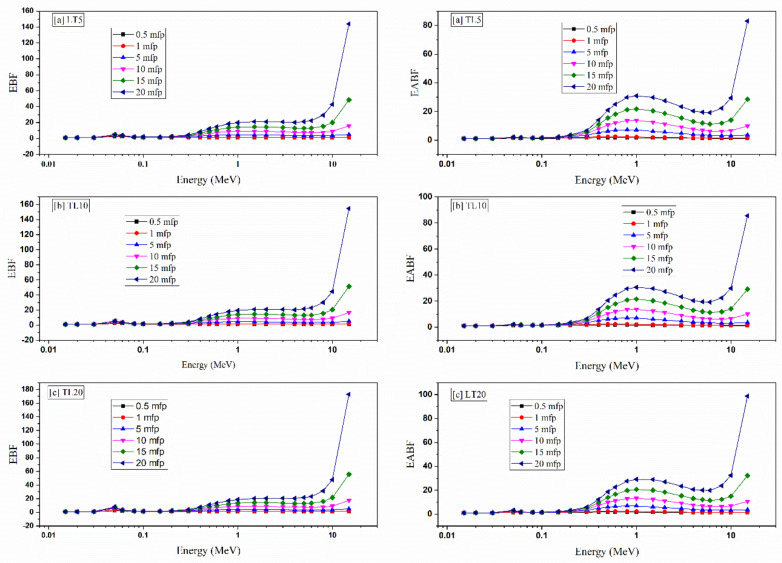
The exposure buildup factor (EBF) and energy absorption buildup factor (EABF) of the LT glass samples.

Figure 9 presents the EBF and EABF for the LT5-LT20 specimens. The obtained values, as presented in Figure 9, depend on three different parameters. The photon flux energy is the first essential factor. Both buildup factors are small for E < 0.1 MeV. In this region, the photoelectric (PE) interaction is the principal phenomenon. Thus, the absorption of photons causes low EBF and EABF values during photon–glass interactions. As energy increases, the buildup factors begin to gradually increase due to the dwindling influence of the PE interaction and the growth of the Compton scattering (CS) interaction. The calculated buildup factors continue to increase with increasing gamma energy, and the CS interaction becomes more dominant until the maximum values are reached, at around 0.8 MeV for both EBF and EABF. The maximum EBF values are 1.340, 1.678, 4.458, 8.264, 12.523, and 17.171, while the maximum EABF values are 1.702, 2.341, 6.931, 12.862, 19.790, and 27.665 at penetration depths of 0.5, 1, 5, 10, 15, and 20 mfp, respectively. Above 1 MeV, the pair-production interaction begins to consume the photons’ energy in the pair reaction (electron-–ositron) process, slightly reducing the accumulation of photons. For very high photon energies (E > 8 MeV), the EBF and EABF greatly increase, especially for penetration depths >20 mfp and energies E > 10 MeV.

The penetration depth (PD) is the second factor that significantly affects the calculated EBF and EABF values. Figure 8 depicts that EBF and EABF are relatively low at small penetration depths. The values then gradually increase as PD increases until 20 mfp. This is because the incident photons will spend a long time within the glass material, decreasing the number of photons that will penetrate the sample. After 20 mfp, the EBF and EABF values jump to high levels. The photons penetrate through the material with few glass–photon interactions for a lower source–detector distance. As a result, the number of accumulated photons is small. When increasing the PD, the number of absorbed or scattered photons increases, increasing the buildup factors.

The data presented in Figure 9 depict that the glasses’ chemical compositions affect the calculated values. The results proved that the substitution of TeO_2_ by La_2_O_3_ causes more photon accumulation. As a result, the EBF and EABF values increase, causing the values to follow the pattern: EBFLT20 > EBF LT15 > EBF LT10 > EBF LT7 > EBF LT5 and EABFLT20 > EABF LT15 > EABF LT10 > EABF LT7 > EABF LT5.

## 4. Conclusions

We analyzed the radiation attenuation capabilities of lanthanum tellurite glasses with a composition of xLa_2_O_3_+(1 − x)TeO_2_, where x = 0.05, 0.07, 0.1, 0.15, and 0.2 mol%. The *LAC*s results showed that the LT20 had the highest *LAC* at all energies, whereas LT5 had the smallest *LAC*. MCNP5 determined the *MAC*s of the selected samples, and the results were validated by the XCOM database. The percent deviation between the values obtained by the two methods (MCNP5 and XCOM) was less than 3% percent for all the selected samples, proving the accuracy of the MCNP5 simulation. The TF of the glasses was also investigated, and it was found that TF greatly decreased as thickness increased. For example, it was observed that when the thickness of the LT5 sample increased from 1 to 5 cm, TF decreased from 0.76 to 0.25 (at 1.332 MeV). The Z_eff_ and Z_eq_ for the studied glass samples were also reported. Moreover, the influence of the energy and the composition of the glasses on these two parameters was discussed. Additionally, the buildup factors of the glasses were calculated, and it was found that the EBF follows the trend: EBFLT20 > EBF LT15 > EBF LT10 > EBF LT7 > EBF LT5. The same trend was also reported for the EABF. This research concluded that the LT20 sample has the greatest potential for radiation shielding applications due to this glass having the most desirable parameters out of the investigated glasses.

## Figures and Tables

**Table 1:** The chemical composition (wt.%) for the LT glass systems.

Glass Code	La_2_O_3_	TeO_2_	Density	Molecular Weight
–	–	–	g cm^−3^	g mol^−1^
LT5	9.7	90.3	5.67	167.91
LT7	13.3	86.7	5.69	171.23
LT10	18.5	81.5	5.7	176.22
LT15	26.5	73.5	5.74	184.53
LT20	33.8	66.2	5.76	192.84

**Table 2:** Comparison between the *MAC* (cm^2^ g^−1^) obtained by MCNP-5 and the *MAC* evaluated via XCOM for 5, 7, and 10 La_2_O_3_ mol %.

Energy	LT5			LT7			LT10		
	MCNP5	XCOM	diff (%)	MCNP5	XCOM	diff (%)	MCNP5	XCOM	diff (%)
0.24	0.23	0.22	3.02	0.23	0.22	2.97	0.23	0.23	2.90
0.66	0.08	0.08	0.30	0.08	0.08	0.30	0.08	0.08	0.30
0.67	0.07	0.08	0.31	0.08	0.08	0.31	0.08	0.08	0.31
1.17	0.05	0.05	1.70	0.05	0.05	1.72	0.05	0.05	1.74
1.33	0.05	0.05	1.42	0.05	0.05	1.42	0.05	0.05	1.43

**Table 3:** Comparison between the *MAC* obtained by MCNP-5 and the *MAC* evaluated via XCOM for 12.5, 15, and 20 La_2_O_3_ mol%.

Energy	LT12.5			LT15			LT20		
	MCNP5	XCOM	diff (%)	MCNP5	XCOM	diff (%)	MCNP5	XCOM	diff (%)
0.24	0.24	0.23	3.08	0.24	0.23	3.07	0.24	0.23	2.76
0.66	0.08	0.08	0.30	0.08	0.08	0.30	0.08	0.08	0.31
0.67	0.08	0.08	0.32	0.08	0.08	0.31	0.08	0.08	0.31
1.17	0.05	0.05	1.74	0.05	0.05	1.75	0.05	0.05	1.78
1.33	0.05	0.05	1.45	0.05	0.05	1.45	0.05	0.05	1.48

## Data Availability

The data presented in this study are available on request from the corresponding author.

## Abbreviations

LAC	Linear attenuation coefficient	diff (%)	The difference between simulated and computed values of *MAC*
I	Incoming intensity	Z_eff_	Effective atomic number
Io	Transmitted intensity	Z_eq_	Equivalent atomic number
MAC	Mass attenuation coefficient	EBF	Exposure buildup factor
TF	Transmission factor	EABF	Energy absorption buildup factor
HVL	Half-Value layer	PD	Penetration depth
ρ	Density of the examined material		
ΣR	Neutron cross-section		
